# The biofilm matrix protects *Bacillusubtilis* against hydrogen peroxide

**DOI:** 10.1016/j.bioflm.2025.100274

**Published:** 2025-03-13

**Authors:** Erika Muratov, Julian Keilholz, Ákos T. Kovács, Ralf Moeller

**Affiliations:** a German Aerospace Center (DLR e.V.), Institute of Aerospace Medicine, Radiation Biology Department, Aerospace Microbiology Research Group, Linder Hoehe, Cologne (Köln), Germany; b University of Bonn, Institute for Microbiology and Biotechnology, Meckenheimer Allee 168, 53115, Bonn, Germany; c Institute of Biology, Leiden University, Sylviusweg 72, 2333 BE, Leiden, Netherlands

**Keywords:** *B. subtilis*, Biofilm, Hydrogen peroxide, Endospores, Exopolysaccharides, EPS, ROS, Oxidative stress

## Abstract

Biofilms formed by *Bacillus subtilis* confer protection against environmental stressors through extracellular polysaccharides (EPS) and sporulation. This study investigates the roles of these biofilm components in resistance to hydrogen peroxide, a common reactive oxygen species source and disinfectant. Using wild-type and mutant strains deficient in EPS or sporulation, biofilm colonies were cultivated at various maturation stages and exposed to hydrogen peroxide. EPS-deficient biofilms exhibited reduced resilience, particularly in early stages, highlighting the structural and protective importance of the matrix. Mature biofilms demonstrated additional protective mechanisms, potentially involving TasA protein fibers and/or the biofilm surface layer (BslA). In contrast, sporulation showed limited contribution to hydrogen peroxide resistance, as survival was primarily matrix-dependent. These findings underscore the necessity of targeting EPS and other matrix components in anti-biofilm strategies, suggesting that hydrogen peroxide-based disinfection could be enhanced by combining it with complementary sporicidal treatments. This study advances our understanding of biofilm resilience, contributing to the development of more effective sterilization protocols.

## 1 Introduction

The ability of microorganisms to grow either as swarming cells or sessile biofilms on surfaces offers numerous benefits compared with planktonic growth in a liquid medium. Transitioning from the single cell motility through coordinated swarming to the immobilized lifestyle provides a flexible approach for nutrient utilization, a uniform proliferation, and increased resilience to environmental stressors [[1], [2], [3]]. Thus, living in multicellular and multispecies communities is the most common form of microbial life, which is highlighted by their ubiquitous occurrence in medical, environmental and industrial settings [4,5]. The high impact of biofilms in these fields, notably within clinical environments, is underscored by statistics released by the National Institute of Health (NIH), revealing that biofilm-associated bacteria are responsible for 60 % of all bacterial infections in humans. Furthermore, biofilms account for 80 % of chronic and 65 % of all nosocomial infections [6,7]. Besides the health aspect, biofilms have a high economic relevance. According to a comparative study from 2022, it is estimated that biofilms have an economic impact about USD 5000 billion per year. The values refer to data from 2019, whereby the majority of the costs were caused by corrosive biofilms in industrial facilities [8]. These findings clearly illustrate the seriousness of biofilms as a health threat and the considerable challenge in inactivating them and developing anti-biofilm strategies. The increased resistance against eradication agents can be attributed to at least four categories [9]:

(I) Slow growth: Similar to the stationary phase in unicellular lifestyles, biofilms undergo physiological adaptations due to slower nutrient diffusion, resulting in reduced metabolic activity [4,10]. This adjusted growth kinetics produce more persistent cells with decreased susceptibility to sterilization regimes and antibiotics that target rapid cell growth [11,12].
(II) Communication: The capability to induce biofilm formation is highly dependent on an efficient cell-to-cell communication, termed quorum sensing (QS). QS systems differ between Gram-positive and negative bacteria and are based on the release of chemical signals [13]. Once these signals are recognized, they can be utilized for optimal environmental adaptation. Hence, QS facilitates efficient nutrient utilization and storage, genetic material transfer, and division of labor. This division of labor triggers cell differentiation, encompassing motility, secondary metabolite synthesis, and production of protective biofilm matrix components [14,15,16].
(III) Extracellular matrix (EM): A key factor in enhancing resistance in biofilms is the protective extracellular matrix produced by the inhabiting cells, which they produce autonomously and occupy [17]. This complex matrix comprises biopolymers, such as extracellular polysaccharides (exopolysaccharides = EPS), proteins, lipids, and nucleic acids [18]. The precise composition of the EM varies among species and is dependent on cultivation conditions, substrates and medium [4,19]. It is assumed that the EM is primarily responsible for the resistance to disinfectants and antibiotics, as it prevents penetration either by adsorption or by reacting with the polymers in the EM [20,21].
(IV) Unknown factors: In addition to I-III, there must be further protective factors. For example, EPS are crucial but not essential for biofilm formation and survival [22,23]. Identifying these protective factors could be challenging due to the dynamic nature of biofilms, which is influenced by various compounds and mechanisms.

To maintain hygienic standards which are necessary to enhance human health but also to reduce costs, effective interventions are necessary to minimize potential risks of infections [[24], [25], [26]]. Such interventions include the disinfection of contaminated surfaces with hydrogen peroxide-based chemicals, a registered disinfectant with bactericidal, viricidal, sporicidal and fungicidal properties [25,27]. Hydrogen peroxide (H_2_O_2_) is classified as one of the reactive oxygen species (ROS), which can arise from intracellular or extracellular oxidizing events, such as radiation exposure or mitochondrial phosphorylation [28]. H_2_O_2_ is a robust oxidizing agent that in the presence of Fe^2+^ generates highly reactive hydroxyl radicals (˙OH) which are able to damage macromolecules, such as DNA, lipids of the cell membrane, and proteins [[28], [29], [30]]. The imbalance between ROS and protective endogenous compartments results in oxidative stress which subsequently cause cell death [31].

*Bacillus subtilis,* a Gram-positive facultative anaerobic soil bacterium, forms complex biofilm consortia and exhibits remarkable resistance owing to its ability of sporulation [32]. Thus, this species is commonly utilized as a biological indicator in decontamination studies [33]. In addition to endospore (hereafter referred to as spores) formation, the multicellular lifestyle offers numerous potential protective properties. The EM primarily consists of exopolysaccharides (EPS) and the protein TasA, which forms amyloid fibers essential for the biofilm scaffold. Moreover, *B. subtilis* biofilms produce a hydrophobin protein coat, formed by BslA, crucial for overall protection against desiccation and selective permeability [[34], [35], [36]]. The organization of this biofilm assembly relies on nutrient availability and extracellular signals [[37], [38], [39]]. Upon signal recognition and adequate environmental conditions, part of the population start to express biofilm-related genes leading to phenotypic heterogeneity that allows coexistence of motile and matrix-producing cells, as well as development of highly resistant spores at the later stage that are assumed to contribute to dispersal [[40], [41], [42]]. This division of labor is tightly regulated and dynamic, with gene expression profiles adapting to environmental conditions [4,43]. These properties demonstrate the remarkable adaptation of biofilms to environmental stressors, making them challenging to inactivate once formed. *B. subtilis* biofilms have been utilized to dissect the influence of various treatment strategies, including disinfection agents, nanoparticles, and laser irradiation [[44], [45], [46], [47], [48]].

This study seeks to elucidate the impact of hydrogen peroxide on bacterial biofilms lacking EPS and spores, thus contributing to the development of targeted strategies for biofilm control and disinfection. Here, we used the architecturally complex colonies of *B. subtilis* to evaluate the role of these protective structures.

## 2 Material and methods

The biofilm cultivation was initiated with spores due to their consistency and stability and the inoculant. Their metabolic inactivity ensures a uniform starting point, preventing variations in metabolic states.

### 2.1 Spore production and purification

For spore production, 200 μL of an overnight culture were inoculated onto solidified Schaeffer sporulation medium (SSM) [49]. The strains used in this study are listed in Table 1. Plates were incubated for 5–7 days at 37 °C to achieve optimal spore quality and quantity. Spores were harvested from the plate using an inoculation loop and resuspended in 40 mL _dd_H_2_O containing sterile glass beads with a size of 3 mm in diameter. This facilitated resuspension using vortexing (2 min) and aided in dispersing cell debris released from the lysed mother cells. To achieve high spore quality and purity, the suspension was repeatedly washed until purity of >99 % spores was confirmed using phase contrast microscopy. The pure spore solution was then stored in glass tubes at 4 °C until utilized.

**Table 1:** *B. subtilis* strains tested in survival ability to hydrogen peroxide. Tet^R^-tetracycline resistance, Cat^R^-chloramphenicol resistance.

**Strain**	**Genotype**	**Deficiency**	**Reference**
**NCIB3610**	Wild type	None	(38)
**ZK3660**	*ΔepsA-O*::Tet^R^	No production of exopolysaccharides within the EM. Remaining matrix components are accomplished by TasA and BslA.	(50)
**F-030**	*comI*^Q12L^*sigG*::Cat^R^	Deficiency in sporulation (inhibition of late forespore polymerase activities)	(51)

### 2.2 Bacterial biofilm cultivation

To obtain biofilms which are standardized and reproducible, a cultivation method according to Fuchs et al. was conducted [52]. Briefly, an inoculum of spores with 10^8^ Colony Forming Units (CFU) mL^−1^ was utilized and pipetted in the middle of a hydrophilized PTFE filter (polytetrafluoroethylene, Merck Millipore®, pore size 0.4 μM, Merck KGaA, Darmstadt, Germany) with a diameter of 30 mm. This PTFE filter separates the growing biofilm physically from the medium, while enabling water and nutrient diffusion. The inoculated filter material was air dried under sterile conditions for 10 min and placed on solidified minimal medium (MSgg), adapted after Branda et al. [38,53]. As *B. subtilis* biofilms are highly heterogenous populations with changing cell and EM profile over time, differently matured biofilms were tested here, ranging from 24 h to 72 h. For 0 h, pure inoculum of spores was pipetted onto the filter material. Here, the treatment was performed directly after the drying process. For the sporulation deficient strain of *B. subtilis* (Δ*sigG*) an overnight culture with planktonic cells from stationary phase with 10^8^ CFU mL^−1^ was used as inoculum and 0 h control.

### 2.3 Sample treatment and CFU determination

Biofilms were grown to distinct development stages and exposed to H_2_O_2_. PTFE filters carrying the biofilms were placed into sterile six-well plates. ROS stress was induced by adding 910 μL of 3 % H_2_O_2_, diluted in PBS, to each biofilm. After treatment durations of 0, 10, 20, 40, and 60 min, the reaction was stopped by adding 10 mg mL^−1^ catalase solution. The treated biofilms were transferred into 2 mL reaction tubes containing glass beads (3 mm diameter). To ensure a reliable yield of viable cells, the reaction mixture suspension was also transferred to the 2 mL tube containing the biofilm. Each tube was vortexed for 2 min and survivability was assessed by calculating total CFU (compromises vegetative cells and spores) and the number of spores. To quantify the spores count, an aliquot of the sample was treated at 80 °C for 10 min to inactivate vegetative cells.

### 2.4 Statistical analysis

The CFU of the total cell mass and spores within the biofilm were calculated at each treatment point of the stress assays as well as the untreated control. Therefore, a dilution series was prepared and plated on LB agar. The average CFU was determined while all data are presented as the average of three biological replicates (n = 3) with according standard deviations. The statistical analysis has been performed by using Tukey's test with SigmaPlot (version 14.5) and OriginLab (version 2023).

## 3 Results

**Fig. 1:**
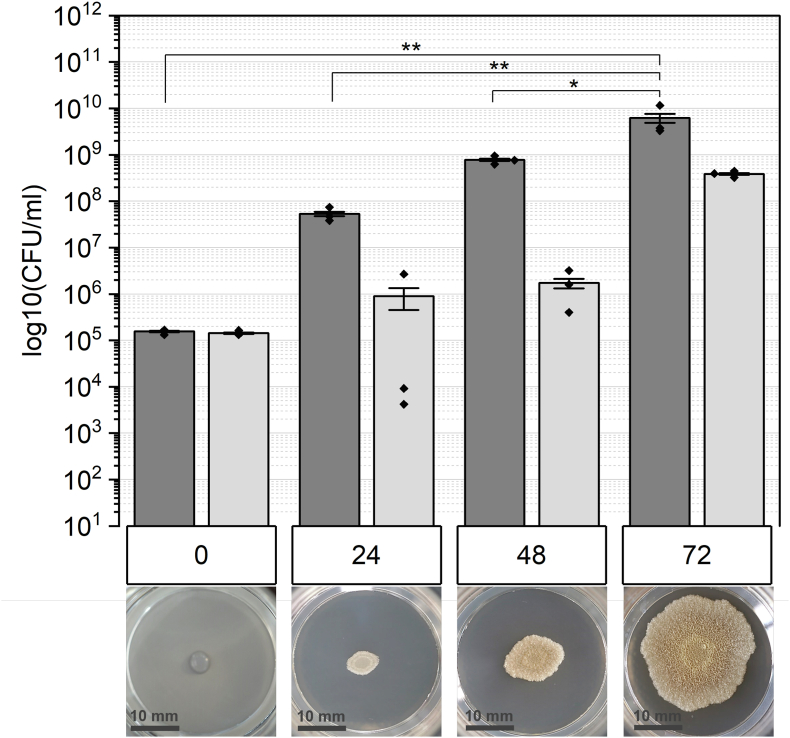
The quantity of untreated wild-type (wt) biofilms is depicted according to each stage of biofilm maturation (in hours). The biofilms are composed of a mixture of vegetative cells and spores, termed as “CFU total” (dark grey bars). Additionally, the proportion of spores was quantified and is represented as light grey bars. The macroscopic morphology is illustrated below for each respective time point.

Hydrogen peroxide is known to be an efficient antimicrobial agent and is commercially used as a disinfectant. Numerous studies have demonstrated its efficacy in combating biofilms formed by *Pseudomonas aeruginosa* and *Staphylococcus aureus*, targeting both the matrix and the cells [25,54,55]. In this study, our objective was to assess the anti-biofilm activity of hydrogen peroxide against *B*. *subtilis* biofilms and the contribution of EPS and spores on survivability. Hence, macrocolony biofilms were cultivated at various growth stages, exposed to hydrogen peroxide and quantified via CFU determination. The cell count within wild-type biofilms consistently rises as maturity progresses, while total CFU comprises a mixture of vegetative cells and spores (Fig. 1). The inoculum (0 h) includes around 10^5^ spores per ml, resulting in little difference in cell count between total CFU and spores at this time point. Spore count peaks in mature (72 h) biofilms, contributing to a notably reduced ratio of vegetative cells. The morphology of wt biofilms varies also with age. In the 24 h stage, the characteristic concentric rings develop. Matured biofilms exhibit increased wrinkling and size. Overall, particularly at 48 and 72 h, biofilms appear as highly heterogenous 3-dimensional structures.

**Fig. 2:**
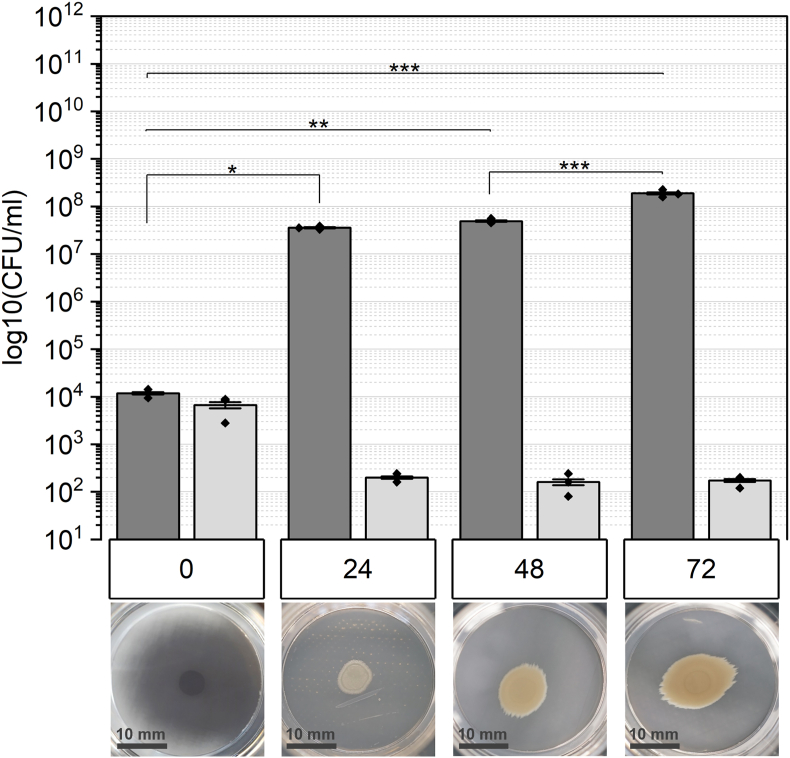
The number of untreated biofilms lacking exopolysaccharides (Δ*epsA-O*) is illustrated for each stage of biofilm maturation (in hours). Dark grey bars represent the total colony-forming units (CFU), while light grey bars indicate the spore count. Statistical significance was determined using Tukey's test with a sample size of n = 3 and indicated by p-values: ∗ <0.05, ∗∗<0.01, ∗∗∗ <0.001.

*B*. *subtilis* biofilms lacking extracellular polysaccharides within the matrix demonstrate variations in cell count when compared to the wt. Initially, at the 0 h timepoint, CFU total and spores exhibit similarity, approximately 10^4^ CFU ml^−1^. However, after 24 h, this pattern reverses, with spore count lower than the inoculum and maintaining consistency over time. Meanwhile, the number of vegetative cells experiences a remarkable increase, approximately fivefold higher than the spore count. Additionally, the lack of EPS leads to a noticeably altered biofilm morphology (Fig. 2). The size of (matured) biofilms remains smaller and exhibits a more uniform structure without wrinkles, showing only one visible concentric ring. Overall, biofilms lacking eps appear less dense and thinner compared to wt.

**Fig. 3:**
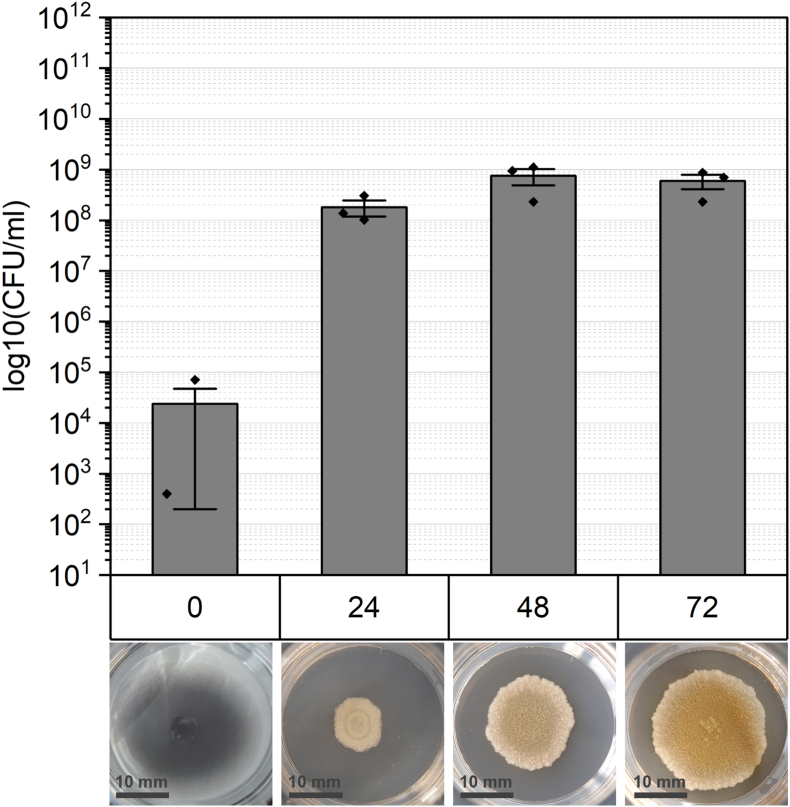
The quantity of untreated biofilms lacking spores (Δ*sigG*) is depicted for each stage of biofilm maturation (in hours). The bars represent only vegetative cells and is termed as “CFU total”. Macroscopic variation among biofilm age is shown below.

*B. subtilis* cell aggregates devoid of spores can be attributed to a deletion in the gene encoding the sigma factor G (Table 1, [51]. Starting from 0 h, with approximately ∼10^4^ planktonic cells per ml, the biofilm expands to double this amount at mature levels. Once a certain threshold is reached, the cell count stabilizes with minimal further increase (Fig. 3).

**Fig. 4:**
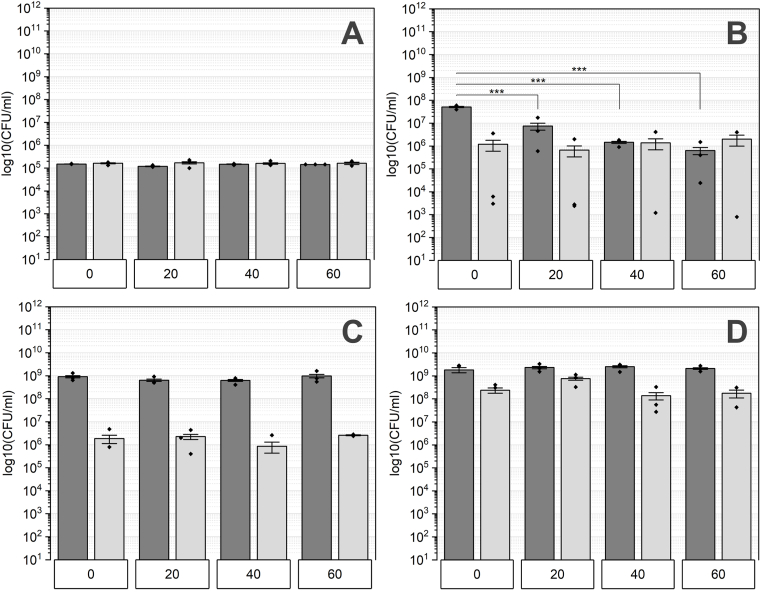
The quantity of wt biofilms treated with hydrogen peroxide is analyzed based on their maturation levels, represented as A: 0 h, B: 24 h, C: 48 h, and D: 72 h. Exposure durations to H_2_O_2_ include 0, 20, 40, and 60 min. Total colony-forming units (CFU) are represented by dark grey bars, while the count of spores is illustrated by light grey bars. Statistical significances were determined utilizing Tukey's test with a sample size of n = 3 and indicated by p-values: ∗ <0.05, ∗∗<0.01, ∗∗∗ <0.001.

Wt biofilms treated with hydrogen peroxide were recovered, and their survival was assessed via CFU determination (Fig. 4). The 0 h timepoint (Fig. 4 0 h) indicates the initial spore count and represents the inoculum reference. Regardless the incubation time, the CFU remains stable, with nearly identical quantities observed between spores and CFU total. Remarkably, even after a 60-min exposure, spore survival remained unaffected. 24 h old consortia showed slight impact in survival and a significant decrease among CFU total, while the spore count was unaffected (Fig. 4 24 h).

However, the spore count remained nearly the same, while for 40 and 60 min treated biofilms, the spore quantity was similar to those of CFU total. Mature wt biofilms, cultivated for 48 and 72 h, exhibited efficient resistance to hydrogen peroxide treatment, as the cell quantity was unchanged throughout incubation time. The only distinction between these maturation stages lies in the higher spore count observed in the 72 h biofilms compared to those cultivated for 48 h.

**Fig. 5:**
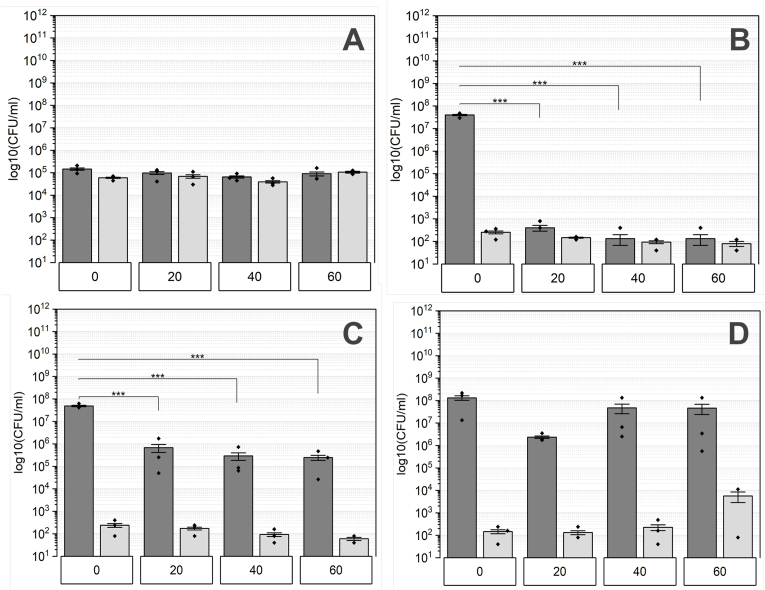
The *eps* deficient biofilms' quantity treated with hydrogen peroxide was assessed at various maturation stages labeled as A: 0 h, B: 24 h, C: 48 h, and D: 72 h. These biofilms underwent exposure to H_2_O_2_ for durations of 0, 20, 40, and 60 min. Total CFU is depicted by dark grey bars, while the number of spores is shown by light grey bars. Statistical significance was evaluated through Tukey's test with a sample size of n = 3 and indicated by p-values: ∗ <0.05, ∗∗<0.01, ∗∗∗ <0.001.

The control inoculum of the *eps* deficient strain shows similar outcomes to those of the WT strain at 0 h and maintains a consistent cell count regardless of the exposure time (Fig. 5 0 h). Following biofilm formation, a mixture of vegetative cells and spores is present, with the spore count in this strain considerably lower than that in the WT strain, as observed in untreated samples. In early-stage biofilms (Fig. 524 h**)**, the total CFU is roughly three times higher than the spore count at the 0-min mark.

However, for exposure durations between 20 and 60 min, the total CFU significantly declines, reaching levels comparable to those of spores.

The subsequent maturation stage, 48-h-old biofilms show a higher survival rate compared to those aged 24 h (Fig. 5 48 h). Nevertheless, a significant reduction in total CFU persists in comparison to the 0-min control. Additionally, there is a slight decrease in the spore count following a 60-min exposure to hydrogen peroxide. Biofilms grown for 72 h show increased susceptibility to hydrogen peroxide after a 20-min treatment compared to those exposed for 40 and 60 min (Fig. 5 72 h). Moreover, the total CFU count slightly increases with longer treatment durations, reaching its peak spore count at the 60-min mark.

**Fig. 6:**
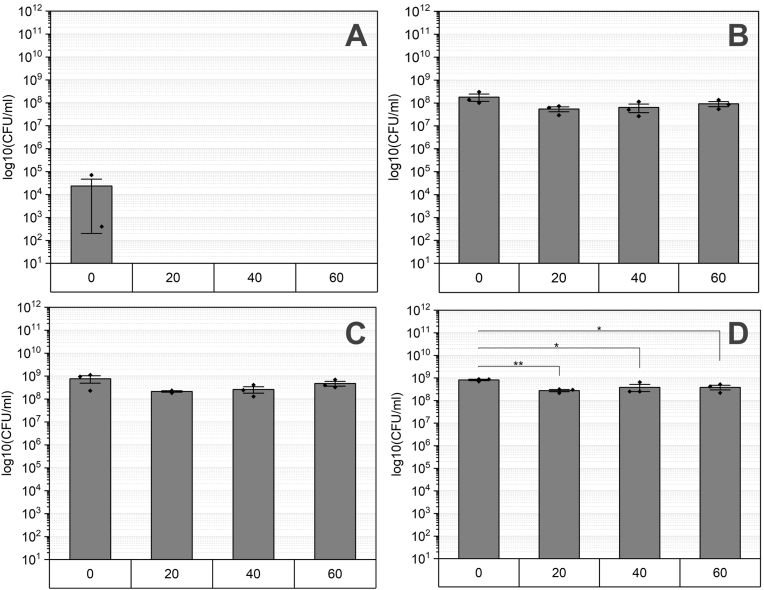
Biofilms deficient in *sigG* were exposed to hydrogen peroxide and tested in survival across different maturation stages labeled as A: 0 h, B: 24 h, C: 48 h, and D: 72 h. Exposure durations to H_2_O_2_ ranged from 0 to 60 min. Statistical significance was analyzed using Tukey's test with a sample size of n = 3 and indicated by p-values: ∗ <0.05, ∗∗<0.01, ∗∗∗ <0.001.

The final strain tested for resistance to hydrogen peroxide lacked SigG, making it incapable of producing spores (Fig. 6). Planktonic cells from the stationary phase were used as inoculum (0 h, Fig. 6 0 h) to evaluate the survival ability by determining the total CFU. After 20 min of treatment, no CFU could be detected and this remained consistent for longer incubation periods. Accordingly, the inoculum of this strain can be regarded as a positive control. Interestingly, once consortia are formed, the cells showed an improved resilience against hydrogen peroxide. Biofilms aged from 24 to 72 h, showed similar results and were only slightly affected (Fig. 6 24 h–72 h). Exposure for 20 min resulted in minimal reduction in CFU, which was constant for longer incubation time.

## 4 Discussion

Hydrogen peroxide serves as a widely used commercial disinfectant, capable of targeting a broad spectrum of microbes, including spores. Some studies even report about its effectiveness against biofilms. However, there is a lack of available data regarding *B*. *subtilis* biofilms and the contributions of EPS and spores to resistance. Moreover, many studies overlook the impact of varying biofilm ages, which could be pivotal in understanding resistance mechanisms. Before the survival assay was conducted, the biofilms in maturation stages ranging from 0 to 72 h were cultivated and analyzed in their morphological phenotype as well as cell and spore quantity.

### 4.1 Comparison of the morphology and cell/spore quantity in wildtype biofilms versus EPS- and spore-lacking variants

The formation of architecturally complex structures as biofilms is attributed to a spatiotemporal cycle involving alternating phases of motile swarming and sessile matrix production [56,57]. This process results in the formation of concentric rings, as observed in wt biofilms, while being less abundant in *eps-*deficient and Δ*sigG* colony biofilms (Fig. 1, Fig. 2, Fig. 3 and [58]). Overall, biofilms lacking EPS show observable differences in texture and size. These consortia are impacted in their physical integrity and display a more homogenous appearance compared to wt. Although there are other matrix structures besides EPS, no wrinkles are visible. This effect strongly suggests that the formation of wrinkles is dependent on all matrix structures [59]. The vertical expansion, or the consolidation phase of biofilms is facilitated by matrix-producing cells, which are disrupted in the *epsA-O*-deficient strain, leading to the thin colony morphology [56]. Nevertheless, the preserved size in early-stage biofilms is maintained by swarming cells, which support two-dimensional expansion, or migration phase. Thus, the overexpression of the motile cell phenotype could compensate for the absence of EPS, thereby aiding in the growth of biofilms (57, Fig. 1 vs Fig. 3). The spatial arrangement of *B. subtilis* biofilms at specific time intervals prompts cell differentiation, including sporulation, at different stages and locations, culminating in characteristic colony morphology [60]. Sporulation seems to be associated with the development of complex architectural formations, as indicated by the diverse biofilm structure observed in our investigation. Furthermore, Aguilar et al. validated the correlation between sporulation and matrix production by the protein KinD [61]. Interestingly, Vlamakis et al. discovered that biofilms lacking spores due to *sigF* deletion do not undergo changes in biofilm structure but reduced spore quantity when matrix production is shut down [42,50].

In our study biofilms which lack EPS in the matrix show as well reduced levels of spores compared to wt (Fig. 2). The cell differentiation within biofilms is a highly regulated process, with matrix production and sporulation being connected through the activity of the bifunctional protein KinD. KinD mediates the phosphorylation (or dephosphorylation) of the master transcription factor Spo0A. As low quantities of phosphorylated Spo0A induces expression of matrix genes, EPS-deficient mutants exhibit delayed sporulation, leading to low spore count [61].

Comparing the overall cell numbers between the investigated strains, wt biofilms tend to an exponential increase in cell number, while the cell quantity of mutant strains reaches a plateau in mature stages. Alterations in nutrient storage and requirements resulting from a disrupted or altered matrix could influence cell and spore numbers, as well as morphology [59,62,63]In addition to the storage and transport, EPS contributes to quorum sensing (QS), which regulates the cell density and expansion of biofilms. The polysaccharides facilitate the stability of signal molecules necessary for QS, enhancing biofilm functionality and maintenance [64].

### 4.2 Contribution of extracellular polysaccharides to survival to hydrogen peroxide

The resistance of cells in a biofilm to a variety of disinfectants and antibiotics is attributed to the protective EM [65]. The matrix of *B. subtilis* biofilms is mainly composed of polysaccharides and proteins and organized in a mobile framework, interspersed with rigid aggregates of cells, Extracellular polysaccharides are more abundant in the mobile section, while protein fibers maintain rigidity [19,66]. Because of the multiple functions of EPS within the matrix, its protective capability against hydrogen peroxide was tested. *The eps* mutants are more susceptible to hydrogen peroxide than the wild type, especially in the early stages of maturation (24 h). Once a certain threshold of cell density is reached (at 72 h), survival is similar (Fig. 5B–D). Only spores (0 h) demonstrated a similar resistance and were not affected in terms of survival (Fig. 5). Interestingly, the resistance characteristics differ across the maturation stages tested. In young biofilms (24 h) only spores survived the hydrogen peroxide exposure, whereas mature biofilms did not exhibit the same resistance. Treated 48 h and 72 h biofilms show a higher number of total CFU than CFU of spores, indicating the survival of vegetative cells. Thus, apart from EPS, additional components within the matrix must contribute to the protection. The structural integrity of biofilms is crucial for surviving harsh environmental stressors, such as reactive oxygen species induced by hydrogen peroxide. Although the exact composition of the matrix varies depending on numerous factors and differs even among species, EPS and proteins are crucial for this integrity and are therefore highly abundant [19,67]. Besides the structural functionality, these compounds enhance the resistance to biocides. On one hand, the matrix acts as physical barrier against antimicrobial agents like hydrogen peroxide. On the other hand, they can react with them resulting in their depolymerization and thus disruption of aggressive hydroxyl radicals. In addition to polysaccharides, amyloid fibers formed by the TasA protein are known to contribute to the resistance [68]. Because of its characteristic beta-sheet structure, the interaction with antimicrobial agents that could lead to proteolysis is hindered, thereby TasA can be invoked in protection [[68], [69], [70], [71]]. Thus, a potential factor contributing to the survival of EPS-deficient (particularly mature) biofilms could be the presence of TasA fibers, but also the BslA. Branda et al. describes TasA and EPS as the most important and abundant structures in the biofilm matrix [37]. Further testing of a TasA mutant is necessary to shed more light on the role of amyloid fibers in hydrogen peroxide resistance. Overall, EPS are crucial for surviving oxidative stress, although they play a minor role in mature biofilms, as indicated by the slight reduction in cell quantity of biofilms. Hydrogen peroxide serves as a major source of reactive oxygen species (ROS) by generating hydroxyl radicals, which can initiate the depolymerization of EPS through the cleavage of glycosidic bonds [72]. However, our results indicate that survival rates in young biofilms are lower than in mature biofilms (Fig. 4). This finding suggests a correlation between biofilm survival and EPS quantity. A higher EPS content likely enhances resistance by acting as a diffusion barrier, limiting ROS penetration into the biofilm matrix. Additionally, certain molecules or compounds within the EPS may function as radical scavengers, further hindering ROS from reaching deeper layers. Following this hypothesis, ROS would be unable to penetrate the biofilm core, thereby protecting embedded cells. This concept is supported by a study conducted by Stewart et al., which demonstrated that hydrogen peroxide neither effectively penetrates *P. aeruginosa* biofilms nor inactivates them [73].

### 4.3 Role of sporulation in hydrogen peroxide resistance

Cell differentiation within *B*. *subtilis* biofilms is a crucial mechanism for adapting to dynamic environmental changes and stressors. This differentiation includes the formation of endospores, which allows the cells to persist under harsh conditions in a metabolically inactive (or reduced) state [74,75]. The transition into this dormant state is triggered by nutrient depletion and is accomplished by a range of different resistance mechanisms [76,77]. This is confirmed by the spore counts shown in Fig. 1 as biofilms mature and nutrient levels decrease. The survival of oxidative stress induced by hydrogen peroxide is ensured by enzymes such as catalases or superoxide dismutase localized in the spore coat [78]. For instance, spore-specific catalases like KatX are crucial for surviving hydrogen peroxide exposure during spore germination [79]. Additionally, we observed macroscopic differences in biofilm morphology appeared between sporulation-deficient populations and wt biofilms (Fig. 1 vs Fig. 3). This observation indicates that the differentiation into spores could contribute to the structural integrity and thus, to biofilm resilience against hydrogen peroxide [80,81]. Hu et al. investigated the resistance of spores and vegetative cells from biofilms of *Clostridium perfringens* to oxidative stress and confirmed that spores were more resistant than vegetative cells and the sessile lifestyle has an enhanced resilience [82]. However, numerous studies report the efficacy of hydrogen peroxide as sporicidal agent. Indeed, Sawale et al. determined D-values ranging from 0.08 to 0.95 min by using concentrations from 22 to 33 %. In our study, wt spores were not reduced after 60 min treatment, but the used concentration was more than ten times lower (Fig. 4 0 h). Using a similar concentration of H_2_O_2_, spores show decreased susceptibly and achieve “hardly any inactivation”,as confirmed by further studies [83,84]. Either increasing the incubation time or concentration of hydrogen peroxide could improve the sporicidal efficacy. Looking on multicellular lifestyle it was expected that based on the spore-specific protection mechanisms, spores would contribute to overall resistance to hydrogen peroxide. Interestingly, our results in turn, showed that the formation of spores did not contribute to the survival rate of biofilms (Fig. 6). While spore formation may not be the primary protective mechanism of the biofilms used in our study, spore formation contributes to protection of *B. subtilis* from hydrogen peroxide in planktonic cultures. The quantity of cells was regardless the maturation or incubation time not affected in survival which emphasizes the importance of an intact and functional biofilm matrix.

## 5 Conclusion

This study has shown that EPS in the matrix play a major role in the protection against hydrogen peroxide whereas sporulation does not. A functional structural integrity with intact EPS are even more protective than the ability of forming spores in surviving oxidative stress. Although our experiments clearly demonstrated the protective role of EPS against H_2_O_2_ treatment, these results can only be indirectly compared to those of the sporulation mutant. The comparison is influenced by the use of different inocula for biofilm initiation and the non-isogenic background of the strains. Furthermore, our results have shown that besides the EPS, especially in mature biofilms, additional protective structures remain which could be given by other matrix components, such as TasA fibers or the surface layer protein BslA. This research has revealed that EPS are crucial for surviving H_2_O_2_ exposure and need to be tackled. Newly developed sterilization approaches are often based on hydrogen peroxide and should be combined with additional sporicidal agents like UV or heat.

## CRediT authorship contribution statement

**Erika Muratov:** Writing – original draft, Visualization, Methodology, Investigation, Formal analysis, Data curation, Conceptualization. **Julian Keilholz:** Methodology, Investigation, Formal analysis, Data curation. **Ákos T. Kovács:** Writing – review & editing. **Ralf Moeller:** Writing – review & editing, Supervision, Project administration, Funding acquisition, Conceptualization.

## Declaration of competing interest

The authors declare that they have no known competing financial interests or personal relationships that could have appeared to influence the work reported in this paper.

Given his role as Co-Editor in Chief, Ákos T. Kovács had no involvement in the peer review of this article and has no access to information regarding its peer review. Full responsibility for the editorial process for this article was delegated to Birthe Kjellerup.

## Data Availability

Data will be made available on request.
